# Transitioning Care in Nephropathic Cystinosis: Overcoming Challenges in Young Adults

**DOI:** 10.1016/j.ekir.2024.10.036

**Published:** 2025-03-04

**Authors:** Cybele Ghossein, Laura Nishi

**Affiliations:** 1 Feinberg School of Medicine, Northwestern University, Chicago, Illinois, USA

## Introduction

Nephropathic cystinosis is a rare autosomal recessive lysosomal storage disorder characterized by systemic cystine accumulation and progressive tissue and organ damage. Earlier diagnosis, kidney transplantation, and cysteamine therapy have shifted the natural history from a fatal pediatric renal disorder to a chronic multiorgan condition.^1^ However, new challenges have emerged around the transition from pediatric to adult care and ongoing multidisciplinary management, including fragmented coordination, decreased patient engagement, and worsening extrarenal outcomes.^2^^,^^3^ Here, we present a young adult with cystinosis who transferred to adult nephrology through a specialized transition clinic aimed at overcoming these obstacles.

## Case Presentation

### Diagnosis and Management in Pediatric Care

The patient is a 23-year-old man who was diagnosed with nephropathic cystinosis at the age of 14 months after presenting with vomiting, constipation, and failure to thrive. Diagnosis was confirmed through mixed leukocyte cystine level testing (2.08 nmol ½ cystine/mg protein; normal < 0.2 nmol ½ cystine/mg protein) and genetic analysis. Oral immediate-release cysteamine bitartrate (CYSTAGON; Viatris Inc, Canonsburg, PA) was initiated immediately after diagnosis, and cysteamine eye drops were started when they became commercially available.

During childhood, the patient developed Fanconi syndrome, requiring long-term fluid and electrolyte supplementation. His medical history is also significant for stage 3a chronic kidney disease with microalbuminuria as well as endocrine, ophthalmologic, musculoskeletal, and neurologic manifestations of cystinosis (Table 1). To improve growth trajectory, he was treated with somatropin and reached an adult height of 184 cm.

**Table 1:** Clinical events, evaluations, and treatment history

Cystinosis diagnosis			
Age	Symptoms		Testing
14 mo	• Vomiting • Constipation • Failure to thrive		• WBC cystine level testing • Genetic analysis for CTNS variants (results unavailable)

WBC cystine level testing			
Diagnosis (mixed leukocytes^a^^,^^b^)	Last observed value (granulocytes^b^^,^^c^)		Trends over time

2.08	1.58		Previously stable, with recent elevation around transition to adult care

Clinical assessment			
Laboratory and other parameters (last observed value)	Imaging studies		
• Weight: 87.9 kg • Height: 184 cm • eGFR: 45 ml/min per 1.73 m^2^ • sCr: 2.1 mg/dl • BUN: 26 mg/dl • uACR: 3.2 mg/mmol • TSH: 1.75 mIU/l	Bone age study (aged 16 yrs of age) • Osteopenia; metaphyseal irregularity Lower limb X-ray (aged 17 yrs) • Mild genu valgum of left lower extremity Swallow study (aged 18 yrs) • Laryngeal penetration of thin liquids without aspiration Renal ultrasound (aged 20 yrs) • Increased cortical echogenicity; mild bilateral pelviectasis; 3 mm calculus in right kidney Echocardiography (aged 20 yrs) • No evidence of left ventricular hypertrophy		

Other diagnoses (by organ system)			

Renal • Fanconi syndrome • Stage 3a CKD with microalbuminuria Endocrine • Hypothyroidism Neurologic • ADHD^d^ • Autism spectrum disorder^d^	Ophthalmologic • Corneal cystine crystals • Hyperopic astigmatism • Optic disc elevation • Fusion with defective stereopsis Dermatologic • Acne vulgaris		Musculoskeletal • Distal myopathy • Swallowing dysfunction^d^ • Pectus excavatum • Osteopenia with pathologic bone fracture • External tibial torsion • Genu valgum • Pes planovalgus • Pes equinus^d^

Medications			
Age at cysteamine initiation	Current cysteamine regimen	Other current medications and supplements^f^	

14 mo (after diagnosis)	IR cysteamine 750 mg Q6H^e^	Calcium-cholecalciferol 600 mg-10 mcg QD Calcitriol 0.25 mcg QD Potassium chloride 20 mEq BID Levocarnitine 330 mg BID Chlorophyllin QD	Dapagliflozin 5 mg QD Levothyroxine 50 mcg QD Losartan 100 mg QD Spironolactone 25 mg QD ER methylphenidate 18 mg QD Tretinoin 0.05% cream PRN
In childhood	Cysteamine eye drops 0.37% QID during waking hours		

ADHD, attention-deficit/hyperactivity disorder; BID, twice daily; BSA, body surface area; BUN, blood urea nitrogen; CKD, chronic kidney disease; eGFR, estimated glomerular filtration rate; ER, extended-release; IR, immediate-release; PRN, as needed; Q6H, every 6 hours; QD, once daily; QID, 4 times daily; sCr, serum creatinine; TSH, thyroid-stimulating hormone; uACR, urine albumin-creatinine ratio; WBC, white blood cell.

a Normal mixed leukocyte cystine level in individuals without cystinosis is <0.2 nmol ½ cystine/mg protein.^1^

b Mixed leukocyte and granulocyte cystine level testing are not interchangeable.^4^

c Target granulocyte level is <1.9 nmol ½ cystine/mg protein.^4^

d First diagnosed in adulthood (aged ≥18 yrs).

e The recommended IR cysteamine dosage for patients aged >12 yrs who weigh >50 kg is 2 g/d divided into 4 doses Q6H. If tolerated, the dosage can be adjusted up to a maximum of 1.95 g/m^2^/d to achieve target WBC cystine levels. Based on a calculated BSA of 2.11 m^2^, this patient’s maximum dosage is 4.11 g/d.^5^

f Due to the limited population size, the benefits of many medications have not been specifically evaluated in patients with cystinosis.

### Transition to Adult Care

**Figure 1:**
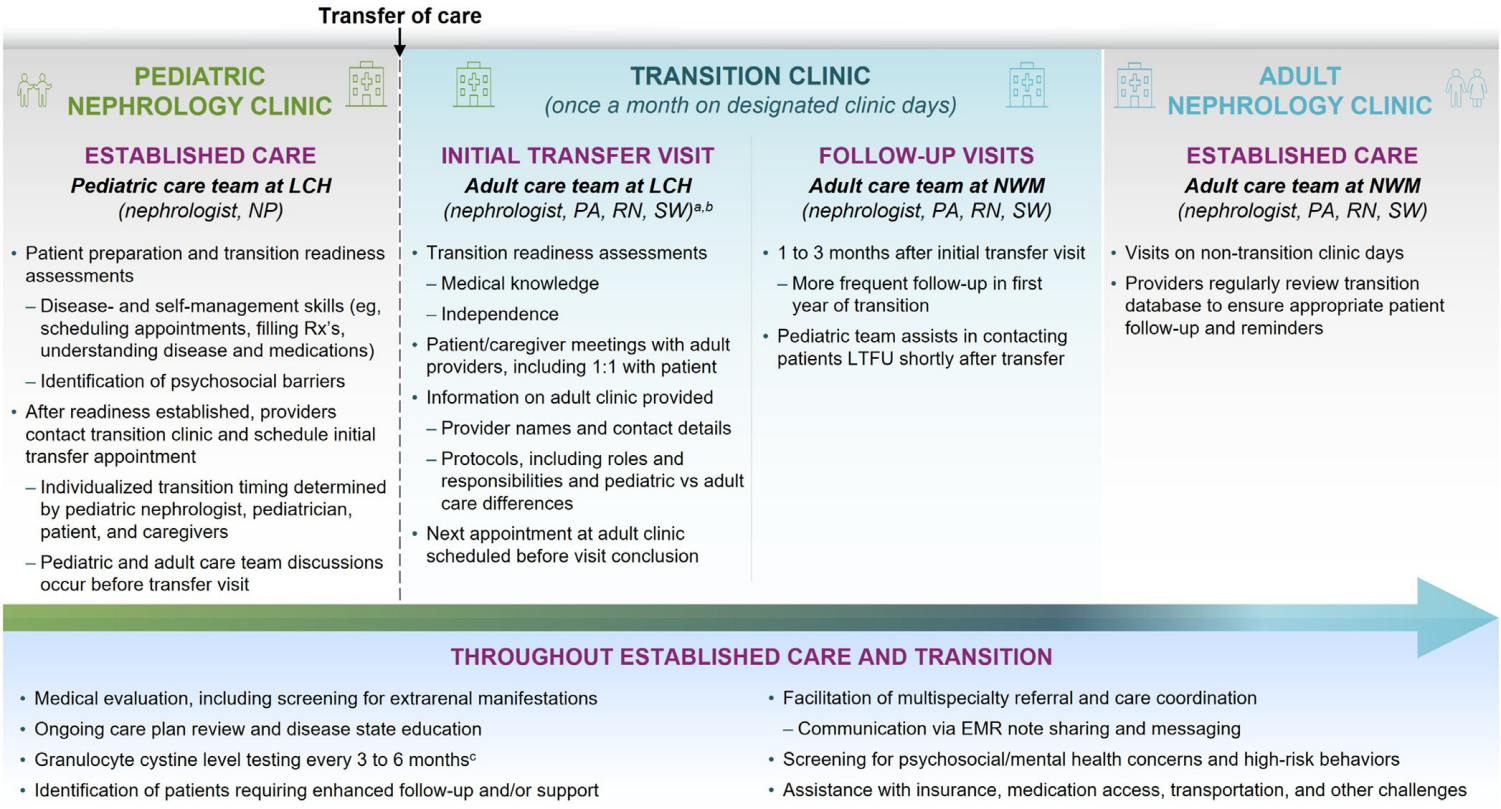
Care model at Lurie Children’s Hospital/Northwestern Medicine for transitioning patients with cystinosis from pediatric to adult nephrology.^6^^,^^7^^a^The adult nephrology transition team is credentialed at the pediatric clinic specifically for this program, facilitating information sharing by allowing providers to access patients’ pediatric medical records. ^b^The pediatric care team is available if needed. ^c^Frequency depends on cysteamine dosage or administration adjustments and/or suspected nonadherence. 1:1, one-to-one; EMR, electronic medical record; LCH, Lurie Children’s Hospital; LTFU, lost to follow-up; NP, nurse practitioner; NWM, Northwestern Medicine; PA, physician associate; RN, nurse; Rx, prescription; SW, social worker.

The patient was referred to our adult nephrology transition clinic by his pediatric team at 22 years of age after considering various factors (Figure 1). He received approximately 1 year of transition preparation, including discussions about overall processes and assessments of disease understanding, medication knowledge, and self-management skills to determine readiness and appropriate timing. Before the initial transfer appointment, pediatric and adult nephrology teams discussed the patient’s pertinent medical history and potential transition barriers, including insurance, transportation, and care engagement. Because the patient was initially diagnosed and managed at another pediatric institution, most of the patient’s early childhood medical records, including white blood cell cystine levels and management approach, were unavailable. A list of formal diagnoses was provided upon referral to his most recent pediatric team.

The transfer visit took place at the usual pediatric site, where the patient met with the adult nephrologist, physician associate, nurse, and social worker alone and with his mother present. Additional information on adult clinic protocols was provided. He attended subsequent visits at the adult facility, and follow-up attendance was monitored over the next year.

## Results

The adult nephrology team sees the patient every 6 months. During recent follow-up, his oral immediate-release cysteamine dosage was increased from 600 to 750 mg every 6 hours due to an elevated granulocyte cystine level of 3.67 nmol ½ cystine/mg protein (goal < 1.9 nmol ½ cystine/mg protein). Repeat testing gradually improved to 2.83 then 1.58 nmol ½ cystine/mg protein. Other laboratory parameters, including kidney function, remain stable, and he takes cysteamine eye drops and medications for renal protection, thyroid replacement, and electrolyte supplementation, among others (Table 1). The patient has not yet required kidney transplantation.

Our nephrology team acts as the “care quarterback” and facilitates as-needed referrals to internal medicine and adult specialties, gauging interest from intrainstitutional providers already caring for patients with similarly complex conditions before consulting others. Multidisciplinary communication typically occurs through electronic medical record messaging or note sharing or phone calls when appropriate. The patient recently reported declining executive function, for which he was referred to neurology and psychiatry. Cognitive and neuropsychological assessments were suggestive of attention-deficit or hyperactivity and autism spectrum disorders, and methylphenidate was prescribed. He has ongoing pain in his feet, and podiatry examination revealed bilateral pronation and equinus deformity. His last ophthalmology evaluation showed prominent bilateral refractile corneal cystine crystals from limbus to limbus, full thickness of deposits in the right eye, and no evidence of retinal crystals.

The patient lives with stable and supportive parents and has recently graduated from college. Our current focus is on regularly monitoring his granulocyte cystine levels to ensure adequate disease control and prevent or delay chronic kidney disease progression and worsening extrarenal complications. Although he has denied significant periods of nonadherence, the patient admits to occasionally missing nighttime cysteamine doses. He remains reluctant to switch to a delayed-release formulation. We continue to emphasize the importance of cysteamine adherence while recognizing that major life changes occurring around the time of the patient’s transition to our clinic likely disrupted medical decision-making and self-management.

## Discussion

**Figure 2:**
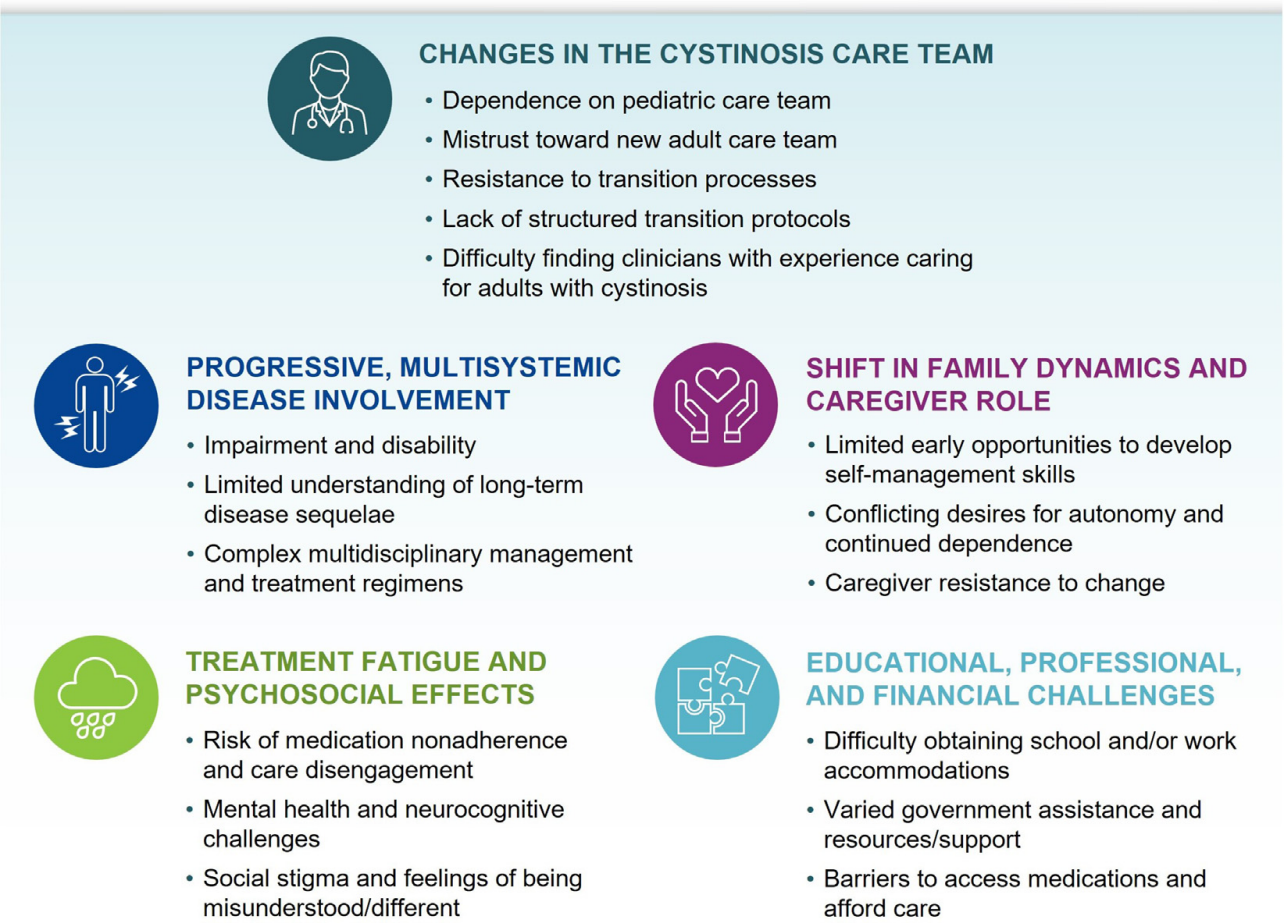
Common challenges throughout the transition to greater independence and self-management in adulthood for patients with cystinosis.^2^^,^^8^

The transition from pediatric to adult care represents a pivotal point in a patient’s cystinosis journey, and insufficient planning can pose significant challenges for patients and clinicians (Figure 2). Care transfer usually occurs between ages of 18 and 24 years during a time replete with greater responsibilities and psychosocial changes.^2^^,^^3^^,^^6^, ^7^, ^8^ Although causes of our patient’s above-target granulocyte cystine levels were not fully discovered, it is not surprising that the elevation coincided with his arrival at our clinic at the age of 22 years, because young adulthood often correlates with reduced medication adherence and care engagement.^2^^,^^8^

Structured transition models, such as that developed by our specialized nephrology clinic, can help facilitate transfer to adult providers, enhance care quality and continuity, and ultimately improve patient outcomes (Figure 1).^2^^,^^5^, ^6^, ^7^, ^8^, ^9^ Transition timing and pace should be tailored to individual patients’ goals and ability to manage care independently.^2^^,^^7^^,^^8^ Establishing a detailed transition plan well before the transfer from pediatric to adult clinicians gives patients time to attain key disease- and self-management skills as well as for the pediatric team to identify and integrate adult providers (Table 2).^2^^,^^8^ Our patient’s transition was preceded by approximately 1 year of education and skill development, followed by coordination among pediatric and adult teams to ensure effective preparation and communication of the patient’s medical history and ongoing needs. Collaboration among clinicians also helps establish trust and rapport with patients. In a 2020 survey assessing perceptions 6 to 12 months after transfer to our adult nephrology clinic (*N* = 42; 40% completed the survey), most patients reported receiving adequate information about transition (100%), feeling comfortable in the adult care setting (94%), and finding value in initially meeting adult providers in the pediatric environment (88%).^7^

**Table 2:** Teaching points

Transitioning to comprehensive adult cystinosis care
• A structured transition plan should be established well before the transfer from pediatric to adult cystinosis care to ensure adequate time to develop patient independence and self-management skills and for clinicians to identify and integrate new providers
• Specialized care models, such as an adult nephrology transition clinic, can help facilitate the transfer of patients with cystinosis from pediatric to adult clinicians and support collaboration among multidisciplinary specialists
• The chronic and complex nature of cystinosis requires a comprehensive multispecialty approach to effectively manage renal and extrarenal manifestations; it is crucial to identify and educate adult providers who are willing to care for patients with complicated medical conditions

Although there are no universally accepted definitions of transition success, the process should ensure uninterrupted care, address evolving medical needs, and support patients’ autonomy and self-advocacy in adulthood.^2^^,^^6^^,^^8^ A 2019 assessment of our transition clinic’s first 3.5 years found that 71% of patients (*n* = 75) successfully transitioned, defined as returning for ≥1 follow-up appointment at the adult nephrology clinic after the transfer visit. Potential factors contributing to our care model’s effectiveness include having a dedicated multidisciplinary transition team, close communication among pediatric and adult providers, increased patient comfort with the transfer visit occurring in a familiar setting, and sufficient 1-on-1 time between patients and clinicians.^6^

Before and after transition, the systemic nature of cystinosis requires a comprehensive approach to limit complications and maintain quality of life. Typically, the pediatric nephrology team acts as the care quarterback and coordinator among multidisciplinary specialists, with responsibilities gradually shifting to adult nephrology and/or internal medicine teams.^2^^,^^3^^,^^8^ However, it can often be challenging to identify adult providers willing to care for patients with complex diseases.^3^ Once multispecialty clinicians are integrated into the team, diligent and proactive communication can ensure continuity of management plans into adulthood.^2^^,^^3^^,^^8^ Our patient has several extrarenal complications that are being monitored and addressed by specialists, and our clinic typically communicates with these providers electronically, referring him to other clinicians and ordering additional evaluations (e.g., swallow study, thyroid level, and pulmonary function tests) as needed.

As the health care system and our understanding of nephropathic cystinosis continue to evolve, efforts to adapt care models to meet young adults’ distinct needs, expand availability of experienced adult specialists, and enhance multidisciplinary coordination are essential to improve outcomes for patients living with this condition. Our patient exemplifies the importance of early transition planning, treatment adherence, as well as individualized and collaborative multispecialty management, as outlined in the teaching points (Table 2).

## Disclosure

CG has received honoraria from Amgen Inc (formerly Horizon Therapeutics plc) for consulting/advisory activities. LN declared no competing interests.

## Patient Consent

The authors declared that they have obtained consent from the patient discussed in this case report.
